# An Automated Method for Rapid Identification of Putative Gene Family Members in Plants

**DOI:** 10.1186/1471-2105-7-S2-S19

**Published:** 2006-09-26

**Authors:** Ronald L Frank, Ajay Mane, Fikret Ercal

**Affiliations:** 1 Biological Sciences Department, University of Missouri-Rolla, Rolla, Missouri, USA; 2 Computer Science Department, University of Missouri-Rolla, Rolla, Missouri, USA

## Abstract

**Background:**

Gene duplication events have played a significant role in genome evolution, particularly in plants. Exhaustive searches for all members of a known gene family as well as the identification of new gene families has become increasingly important. Subfunctionalization via changes in regulatory sequences following duplication (adaptive selection) appears to be a common mechanism of evolution in plants and can be accompanied by purifying selection on the coding region. Such negative selection can be detected by a bias toward synonymous over nonsynonymous substitutions. However, the process of identifying this bias requires many steps usually employing several different software programs. We have simplified the process and significantly shortened the time required by condensing many steps into a few scripts or programs to rapidly identify putative gene family members beginning with a single query sequence.

**Results:**

In this report we 1) describe the software tools (SimESTs, PCAT, and SCAT) developed to automate the gene family identification, 2) demonstrate the validity of the method by correctly identifying 3 of 4 PAL gene family members from Arabidopsis using EST data alone, 3) identify 2 to 6 CAD gene family members from *Glycine max *(previously unidentified), and 4) identify 2 members of a putative *Glycine max *gene family previously unidentified in any plant species.

**Conclusion:**

Gene families in plants, particularly that subset where purifying selection has occurred in the coding region, can be identified quickly and easily by integrating our software tools and commonly available contig assembly and ORF identification programs.

## Background

Ohno described gene duplications and the resulting changes in selective pressure as providing the opportunity for duplicates to evolve new functions in 1970 [1]. Since then it has become clear that a significant proportion of genes that make up a genome are not entirely unique from one another but are part of larger families of related genes. Genome evolution, at least in part, proceeds by duplications of individual genes [2], genomic segments [3], or even whole genomes [4,5]. The accumulation of different mutations in duplicates (paralogs) subsequently leads to either loss of function for one (death), altered function (subfunctionalization), or a new function (neofunctionalization).

The study of the molecular processes by which functional innovation occurs interests not only evolutionary biologists, but protein engineers and agricultural biologists. A clearer understanding of the extent to which gene families contribute to the selected traits in our most important crop species will help guide decisions regarding future improvements.

Many analyses of the divergence between paralogs involve the estimation of the dN/dS ratio, the number of nonsynonymous base substitutions per nonsynonymous site versus the number of synonymous base substitutions per synonymous site [6]. A dN/dS ratio < 1 indicates purifying or negative selection (lower fitness) that tends to keep amino acid sequences the same if changes are deleterious. A ratio equal to 1 indicates changes that are neutral to fitness, while a dN/dS ratio > 1 would indicate adaptive or positive selection presumably because natural selection favors the amino acid changes. Most methods of estimating dN/dS are approximate because many codons may differ in more than one position and therefore could have evolved by different but equally parsimonious paths. Other factors can also make detection of purifying versus adaptive selection difficult, namely codon bias, the fact that selection pressures are not uniform along an evolutionary lineage, and selection is not equal among functional domains of a gene [7].

Force et al. proposed a DDC (duplication-degeneration-complementation) model whereby complementary deleterious mutations in regulatory elements between duplicate genes partitions the original function resulting in different sub-functions [8]. Papp et al. found that the number of shared regulatory elements between duplicated genes in yeast decreases with evolutionary time [9]. Duplicate genes were identified as symmetrical best hits (SymBets) of encoded proteins (E < 10^-20^). The age of the duplicates was estimated by the accumulation of synonymous substitutions in the coding regions. If synonymous substitutions were accompanied by an equal number of nonsynonymous (neutral selection acting on the coding region) or by a greater number of nonsynonymous substitutions (adaptive selection acting on the coding region) then one would not expect that the proteins would maintain enough similarity to be SymBets. It follows that if subfunctionalization is to occur by changes in regulatory elements then some degree of purifying selection should maintain the protein function. Otherwise, the adaptive selection on the regulatory elements would be eliminated by loss of original function in the protein. Coding regions of paralogs that have subfunctionalized via changes to regulatory elements should exhibit a bias toward synonymous substitutions. In plants, a significantly greater proportion of genes belong to gene families than in animals or other major taxa [10]. Either gene duplication events have been more common in plants, or more duplicates have been retained during the evolutionary history of plants. In either case, subfunctionalization via changes in regulatory sequences following duplication appears to be an especially common mechanism of innovative evolution in plants [10]. If this is the case, there should exist a significant number of gene families that can be identified by a bias toward synonymous substitutions when contigs are assembled from a significantly large database of ESTs (Expressed Sequence Tags). ESTs are generated when large numbers of randomly selected cDNA clones from various tissues, genotypes, developmental stages, or treatments are partially sequenced. The greater the number of ESTs from independently constructed libraries the more information can be derived from *in silico *analyses. Thirty species currently have over 100 K ESTs in the NCBI database.

In this paper we outline a simple method to identify, from ESTs, gene families that exhibit purifying selection during subfunctionalization of paralogs. We also describe several software tools developed to automate many of the time-consuming steps in the identification of these gene family members. We demonstrate the validity of the method by identifying, from EST data alone, a well-characterized gene family of Arabidopsis. And finally, we demonstrate the identification of two previously unidentified gene families in *Glycine max*, one a known plant gene family and the other a putative novel small gene family.

## Results and Discussion

### Validation of concept

Since our goal is not detecting rates of synonymous versus nonsynonymous substitutions we simply tally differences between potential paralog pairs with regard to codon position. The rationale is that only 8 of 61 first positions of codons are 2-fold redundant (2 bases encode same amino acid), 0 of 61 second positions are redundant, while 24 of 61 third positions are 2-fold redundant, 3 are 3-fold redundant, and 32 are 4-fold redundant. If differences between contigs are evolutionary and subject to negative selection, significantly more differences will occur in the third position and least will occur in the second position. However, if differences between contigs are artifacts (cDNA cloning, sequencing errors, etc) or the result of alternative splicing, no pattern among codon positions should be exhibited. If differences appear nonrandom with respect to codon position (X^2^,*p *< 0.005), and third position differences are more than 3 times first position differences, and all differences are distributed as 3^rd ^> 1^st ^> 2^nd^, then we conclude that the contigs represent different genes. However, if these criteria are not met we do not conclude that the contigs necessarily represent the same gene. Our goal is only to identify contigs that represent different genes of the same family. We do not expect that all members of a particular family will be detectable by this method. If desired other members may be identified with iterative searches using previously identified contigs.

To illustrate that this method can identify members of a gene family with some accuracy using only EST data we tested it on a well-characterized gene family (PAL, phenylalanine ammonia-lyase) from Arabidopsis.

The SimESTs search of *A. thaliana *dbEST using AtPAL1 protein as query (tBLASTn) resulted in 223 EST files (E < 0.01) in FASTA format. The ESTs assembled into nine contigs ranging from 455 to 1547 bases and 2 to 48 ESTs each. Assembly parameters were 20 base minimum overlap with 100% match, and gap creation and extension penalties set at 10.00 each. The few gaps created were never more than one base. The stringent overlap criteria and gap penalties reflect the fact that we would rather miss a potentially valid contig than generate an invalid one. Most contigs exhibited large open reading frames that extended most of the contig length. Some contigs had shorter overlapping ORFs that could be joined by eliminating a single nucleotide gap. However, no major sequence editing was done to increase the quality of contigs. Twenty-eight pairwise alignments were made and the differences according to codon position reported by SCAT are show in Table 1. The pattern exhibited suggest that AtContig1 represents a different PAL gene than AtContig4. That is, 14(14)4(4)43(43) shows nonrandom distribution of differences (p < 0.000001), third position differences are 3.1-fold greater than first position, and overall 3^rd ^> 1^st ^> 2^nd^. Similarly, AtContig3 and AtContig4 represent different PAL genes by these criteria. The eight contigs assorted into three groups based on their nucleotide substitution pattern with each other contig. Those three groups designated GeneA, GeneB, and GeneC are represented by AtContig1, AtContig3, and AtContig4, respectively. AtContig6 and 8 were included as part of GeneC in the comparison to real sequences because they exhibited close similarity to AtContig4. The results of the comparison to the actual gene sequences for the PAL gene family of Arabidopsis are shown in Table 2. Each contig representing the three gene groups was aligned with each of the four actual PAL gene sequences from Arabidopsis. Each contig group identified, by greater than 96% similarity, a different member of the PAL gene family. Additionally, each contig exhibited less than 86% similarity to other members of the gene family. AtContig1 (GeneA) represents AtPAL1 (AY045919), AtContig3 (GeneB) represents AtPAL4 (AC009400), and AtContig4 represents AtPAL2 (AY133595). No contigs generated at the parameters specified in the assembly program represented AtPAL3. Sequence comparisons between the four actual AtPAL genes show that AtPAL 3 is only about 70% similar to the original query gene, AtPAL1, while AtPAL2 is 90% similar, and AtPAL4 about 80% similar to the original query. That is, AtPAL3 is the most divergent gene family member relative to our query sequence. This may explain the reason contigs representing PAL3 were not generated. This may indicate a lower limit to the similarity necessary between paralogs for identification by this method. Also, a potential upper limit of 95% similarity may be indicated by the fact that AtPAL1, the query sequence, and its contig generated from ESTs are 96% similar. That is, paralogs of very recent divergence or otherwise highly conserved at the nucleotide level may not be resolved as unique by this method. An alternative explanation for missing AtPAL3 could be under representation in the EST database relative to the other genes. Our stringent contig assembly parameters resulted in a significant number (41%) of EST sequences not joining a contig but remaining as singletons.

The overall result from this experiment verifies that it is possible to identify at least some gene family members using EST data alone.

### CAD gene family identification in *Glycine max*

We then turned our attention to the identification of a gene family not yet identified in *Glycine max*. Cinnamyl alcohol dehydrogenase (CAD) is an enzyme of lignin biosynthesis, a component of the cell wall, and is encoded by a nine-member gene family in Arabidopsis. The SimESTs search of *G. max *dbEST using AtCAD protein as query resulted in 250 EST files in FASTA format. The ESTs assembled into 17 contigs ranging from 611 to 1355 bases and 2 to 42 ESTs each. Nine contigs that exhibited nearly full length ORFs were processed by SCAT to yield the results shown in Table 3. The results indicate by our criteria that Contig7 represents a different gene than Contig12, i.e., 8(8)5(5)28(28) shows nonrandom distribution of differences (p < 0.00001), third position differences are 3.5-fold greater than first position, and overall 3^rd ^> 1^st ^> 2^nd^. This result alone would constitute an initial putative identification of a gene family not previously described in *Glycine max*. However, the results generated for Contig5 versus Contig15, and Contig2 versus Contig8 meet two of the three criteria and could warrant further investigation as additional members of this gene family. No significant similarity (NS) indicated by bl2seq between Contig2 and the others could be interpreted in two ways. 1) The two contigs in question could represent significantly different genes, or 2) the contigs could represent non-overlapping regions of the same gene. CAD is a protein of about 360 amino acids in Arabidopsis and our contig ORFs are 200 to 340 codons. Therefore, they should all overlap by a minimum of 40 codons. It is possible that those 40 codons are dissimilar enough to generate no significant similarity. However, if option one is correct, then we have potentially identified six members of the CAD gene family of soybean, if incorrect, at least two. Clearly, this warrants further experimental analysis in the lab. Next we plan to construct gene specific primers for PCR and isolate these members of the CAD gene family from soybean. Additional application of SimESTs and SCAT using the contig sequences as queries could identify more members of this gene family.

There are hundreds of plant gene families that have been characterized in only a handful of species. With this method many of those can now be identified in any species for which a significant number of ESTs exist. Soybean has over 350,000 ESTs in dbEST and relatively few gene families. An obvious next step made possible by rapid screening of SCAT is to search for all known plant gene families not yet identified in soybean.

### Novel gene family identification in *Glycine max*

EST clustering can be a simple and effective method for identifying gene families. UniGene, the most widely available clustered set, uses a build procedure that begins by clustering mRNA sequences. The ESTs are then joined to existing mRNA clusters. New clusters are created for ESTs that do not join an mRNA cluster. Since there are 15,047 clusters in Build #22 and only 742 contain mRNA sequences, most clusters are EST-only. UniGene also indicates for each cluster lacking an identifying mRNA a similarity measure to other known genes, either strongly similar to, moderately similar to, or weakly similar to. However, several clusters are designated only "Transcribed locus" because the ESTs show no significant similarity to any known genes. With the speed of screening provided by SCAT we decided to examine these clusters specifically for evidence that some may contain EST sequences from multiple gene family members. Previous analysis of mRNA-containing UniGene clusters revealed that many clusters contain mRNA sequences from two or more members of a gene family [11]. It follows that many of the EST-only clusters could also contain sequences from multiple genes. The first indication of such a case was found in UniGene cluster Gma.9010 containing 156 ESTs. Assembly resulted in two contigs of 606 and 837 bases, each with two significant open reading frames (one on each complementary strand). Results from pairwise alignments using SCAT are shown in Table 4. Contig1a and 1b represent the two ORFs on the same contig. Since bl2seq will automatically compare reverse complements during alignment results for Contig1a versus Contig2a and Contig1a and Contig2b are identical and exhibit a reverse pattern to Contig1b versus Contig2a and 2b. The results for the latter comparisons exhibit the characteristic pattern of third position bias, however they only border on meeting the three criteria. That is, 6(7)6(7)15(16) shows nonrandom distribution of differences (p < 0.0497), third position differences are 2.5-fold greater than first position, and overall 3^rd ^> 1^st ^= 2^nd^. However, these results also indicate a gap by the numbers in parentheses, and that gap is exactly three nucleotides, one in each position of a codon. Examination of the alignment shows that they are consecutive and represent a single codon indel (data not shown). This additional information taken with the pattern causes us to identify this as a potential gene family for further experimental analysis in the lab.

Without even starting with a query it is possible to identify potential gene families from other groups of sequence-similar ESTs. The next phase being pursued by our group is the rapid screening by SCAT of the largest EST-only UniGene clusters.

## Conclusion

Gene families in plants that exhibit purifying selection between members can be identified quickly and easily from EST databases by integrating software tools SimESTs PCAT, or SCAT with commonly available contig assembly and ORF identification programs. This is illustrated by the successful identification of 3 members of a well-characterized small gene family in Arabidopsis using EST data alone. Two additional approaches with unisolated gene families in *Glycine max *also produced positive results. Orthologous sequences can be used to identify at least some members of previously uncharacterized gene families, and new gene families can be discovered. With this method many gene families can now be identified in any plant species for which a significant number of ESTs exist.

## Methods

### Software implementation

SimESTs, PCAT and SCAT are software applications developed to aid in the gene family member identification process. The applications are developed in perl and can run in Unix, Linux and Windows platforms. They make remote calls to the online tools provided by NCBI. The applications use basic libraries necessary for text processing and remote connection using functions like "http request" and "http response". The inputs to these applications are either accession numbers or the sequences in FASTA format which are submitted over the internet. The speeds of the applications depend on the response time of the online tools provided by the NCBI server.

### SimESTs

Similar ESTs (SimESTs) is a software tool which automates the process of finding ESTs similar to the input sequence. The software tool searches dbEST of NCBI for similar ESTs using the BLAST algorithm. The resulting accession numbers of ESTs are used to extract the ESTs in FASTA format. ESTs are preferred in FASTA format because many software tools including the assembly software tools require the input to be a FASTA sequence.

SimESTs is developed in perl because of the strengths of perl in regular expression matching and remote calls to a web server. The software uses string matching to extract useful information from files and also uses online tools of NCBI such as BLAST and bl2seq [12].

**Figure 1:**
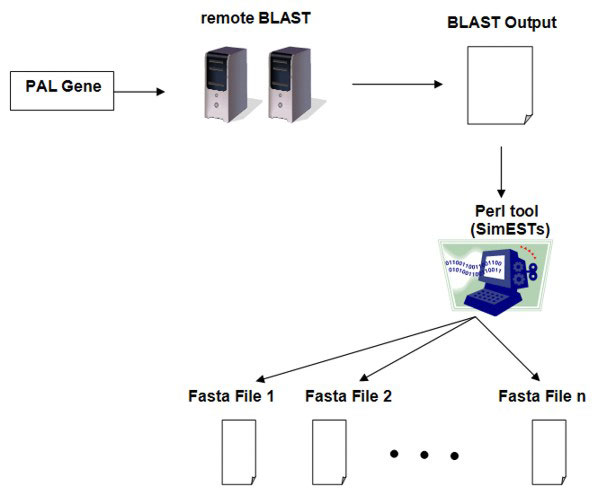
**Overview of SimESTs**. Flow chart of SimESTs input, remote calls, and output.

The input to the software tool is typically an accession number of a known gene family member. When SimESTs is executed, it launches the online tool of NCBI BLAST in the web browser (Figure 1). A user can input the accession number and other parameters and perform the search. The resulting BLAST output is stored in a temporary file which contains the list of accession numbers similar to the query sequence. These accession numbers are followed by the marker "gi" in the file. The file is then parsed using perl's string matching techniques and the accession numbers are extracted. Given a string of characters, the regular expression features of perl can detect if an expression occurs at the start or any other location of a string. The functions "index" and "substring" are used to extract the accession numbers which follow a standard marker.

After the accession numbers are extracted, SimESTs makes remote calls to the NCBI Entrez search tool to extract the ESTs in FASTA format. NCBI provides e-utilities for Entrez tool which can be called from any program on a remote computer. SimESTs uses the efetch tool of e-utilities, which takes an accession number as input and gives the FASTA format file of the EST. A server side program "efetch.fcgi" is executed on the NCBI server that sends ESTs in FASTA format to SimESTs. The first line of the FASTA file has the information about the EST starting with the expression ">". This line is deleted to retain only the sequence which actually represents the EST. All the EST sequences are saved in individual files named for the accession numbers.

SimESTs is a software application which automates all the remote calls to the online tools and processing of data into a single application which takes the accession number as input and produces individual EST files similar to the query in FASTA format. These EST fragments are used later for producing contigs. The speed of the application depends on the number of accession numbers generated and the response time of the Entrez e-tool of NCBI.

### PCAT

Pairwise Contig Alignment Tool (PCAT) is a software tool which aligns two contigs and analyzes their similarity. It calculates the differences at first, second and third positions of the codons of the coding region of the two sequences.

PCAT uses perl to make remote calls to the NCBI online bl2seq tool to align two sequences. The bl2seq program uses the BLAST algorithm to compare two input sequences and displays the alignment and the coding region.

The input to PCAT is a pair of sequences. The sequences can be a combination of accession numbers or actual sequences in text files. Open reading frames of the top sequence of alignment are also input to the software tool. PCAT makes remote calls to bl2seq by invoking the wblast2.cgi program which runs on the web server of NCBI. The server side program takes two sequences and produces the alignment with indices.

The output file is then parsed and the indices of alignment and coding region are extracted using perl's string matching features. The bl2seq alignment start index is listed before the top sequence of the alignment. The exact coding region is calculated based on the relative indices of coding region. These indices are calculated using open reading frames of the top sequence of the two sequences and the indices produced by bl2seq. If there is an overlap of the indices from bl2seq and the open reading frames, then an overlap is reported, otherwise no overlap is reported. In the case of an overlap, the start and end positions of the exact coding region are calculated. This is done by looking for the first position of an open reading frame following the bl2seq start index.

The start and end indices of the exact coding region are used to calculate the differences at the first, second and third positions of all the codons in the coding region. But before this all the gaps in the query sequence are removed with the corresponding bases of the subject sequence. Two scores are calculated for each of the positions of codons. The first score is the mismatch of the bases and the second score is the mismatch of the bases including gaps in the subject sequence. The second score is reported in parentheses following the first score. An example score would be 19(19) 7(7) 70(70) if no gaps are found or 19(20) 7(8) 70(71) if a gap of one codon exists.

Using the PCAT application, accession numbers or the sequences in flat files are submitted over the internet. The sequences in the flat files are extracted using the "concatenation" of the lines of file. Since the input to the application is only a pair of sequences, the speed of the application is only a single call to the bl2seq web tool and the time taken for text processing.

### SCAT

**Figure 2:**
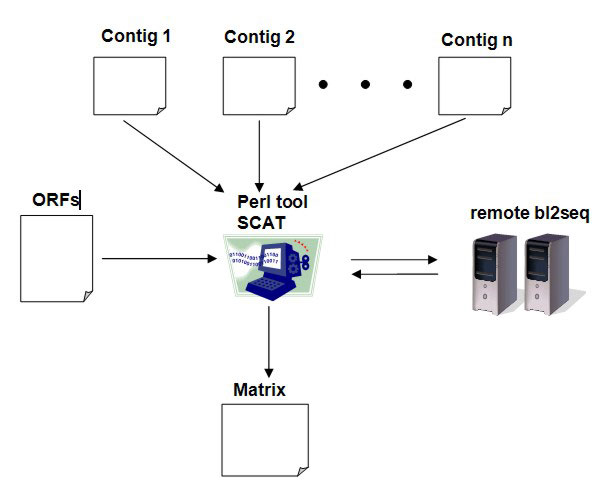
**Overview of SCAT**. Flow chart of SCAT input, remote calls, and output.

Summary Contig Alignment Tool (SCAT) is a software application which automates PCAT for a collection of contigs. PCAT analyzes the similarity of two contigs where as SCAT extends the pairwise alignment to any number of contigs (Figure 2).

The input to SCAT is a file containing the collection of contig filenames and the corresponding open reading frames for each contig. The contig filenames and open reading frames are delimited by a comma. SCAT parses the input file and does a pairwise comparison of all the contigs.

For each pair of contigs SCAT calls the online bl2seq tool remotely. The server side program "wblast2.cgi" takes the two sequences and produces the alignment. The output is then parsed and the coding region and alignment scores are calculated using the similar method used by PCAT. The differences in the positions of the codons are calculated for the coding region.

The pairwise comparisons of all the contigs are automated and summarized in a matrix. The first row and column of the matrix have the contig names and the inner cells of the matrix have the alignment scores. The matrix is usually an upper triangular matrix because reversing the contig order of comparison does not effect the alignment. The lower triangular matrix is filled by asterisks. See Table 1.

SCAT summarizes the similarity of a list of contigs. The input to SCAT is a file containing contigs and the output is a matrix allowing the contigs to be classified into different gene family members. Since the input to the application is a collection of contigs the application speed depends on the number of contigs. The application generates a summary both in an HTML table and a comma delimited file for use in any spreadsheet program.

## Applications to gene family identification

### Overview of procedure

**Figure 3:**
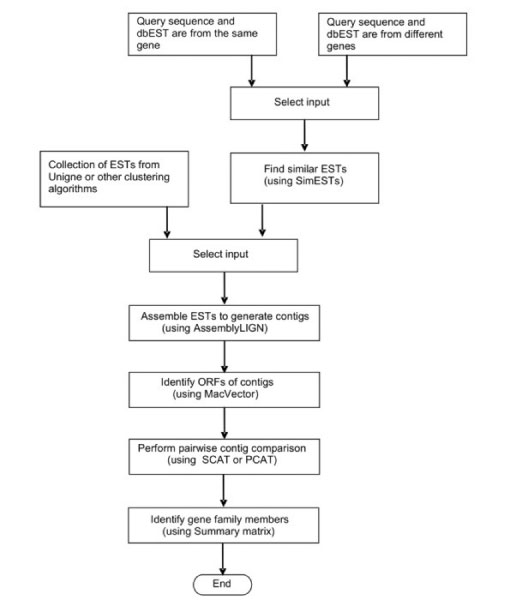
**Flow chart of automation**. Flow chart of all steps in identification of a gene family.

Figure 3 shows an overview of the steps involve in the gene family identification protocol. The starting collection of sequence similar ESTs can be generated by a BLAST search of dbEST (limit by Entrez query [ORGN]) with a protein sequence as query (tblastn). The query sequence can be a known member of the gene family of interest, if available, or an orthologous sequence from a related species. Alternatively, a cluster of ESTs from UniGene or other gene-indexing algorithm such as d2 [13], PECT [14], or PaCE [15].

### Test of concept

As a test of our proposed method of identifying paralogs, one member of the PAL (phenylalanine ammonia-lyase) gene family (4 members) in Arabidopsis (AtPAL1, AAK76593) was used as query in a BLAST search of *A. thaliana *dbEST using software tool SimESTs. The resulting EST sequences in FASTA format were conservatively assembled into contigs (100% match of overlap) using AssemblyLIGN (Oxford Molecular). Open reading frames were identified in each contig using MacVector (Oxford Molecular). The web based program bl2seq [12] was used to align contigs in pairwise fashion while differences between the contigs were tallied as to codon position. All possible pairwise alignments were made using software tool SCAT. The contigs were assorted into groups of putative genes based on judgments made regarding the pattern of nucleotide substitutions. A representative contig from each group was subsequently compared to the actual gene sequences (TAIR, The Arabidopsis Information Resource) to reveal whether or not the EST generated contigs identified a real gene family member, and if so, how similar it was to that gene family member and others.

### *Glycine max *gene family from orthologous query

One member (AAC33211) of the nine-member Arabidopsis CAD (cinnamyl alcohol dehydrogenase) gene family was used as query in a BLAST search of *Glycine max *dbEST using SimESTs. The resulting EST sequences were assembled into contigs, open reading frames identified, aligned in pairwise fashion, and differences tallied as to codon position as described above using SCAT. The contigs were assorted into groups of putative genes based on judgments made regarding the pattern of nucleotide substitutions.

### *Glycine max *gene family from UniGene cluster

The ESTs of UniGene *Glycine max *cluster Gma.9010 were downloaded from NCBI. The EST sequences were assembled into contigs, open reading frames identified, aligned in pairwise fashion, and differences tallied as to codon position as described above using SCAT. The contigs were assorted into groups of putative genes based on judgments made regarding the pattern of nucleotide substitutions.

## Figures and Tables

**Table 1:** Arabidopsis PAL contig comparisons. Tallies of first, second, and third position differences between contigs. Parentheses count gaps as differences. No gaps exist if numbers inside and out are the same. NS means bl2seq returned "no significant similarity." NO means bl2seq returned an alignment but the ORFs did not overlap.

	AtContig3	AtContig4	AtContig5	AtContig6	AtContig7	AtContig8	AtContig9
	
AtContig1	20(20) 6(6) 33(33)	14(14) 4(4) 43(43)	NS	NS	NS	NS	18(18) 5(5) 32(32)
AtContig3	***	45(45) 19(19) 146(146)	NS	19(19) 7(7) 70(70)	NS	15(15) 7(7) 55(55)	5(5) 5(5) 6(6)
AtContig4	***	***	NS	3(5) 4(4) 4(4)	NS	1(2) 2(2) 1(1)	10(10) 4(4) 27(27)
AtContig5	***	***	***	NO	NS	NS	NS
AtContig6	***	***	***	***	6(6) 5(5) 39(39)	4(5) 3(3) 7(7)	NS
AtContig7	***	***	***	***	***	NS	NS
AtContig8	***	***	***	***	***	***	NS

**Table 2:** Arabidopsis PAL contig and gene comparisons. Percent similarity of representative contigs from each grouping to the four actual Arabidopsis PAL gene sequences.

		**GeneA**	**GeneB**	**Gene C**		
		
		Contig1	Contig3	Contig4	Contig6	Contig8
AY045919	AtPAL1	96%	76%	86%	83%	86%
AY133595	AtPAL2	79%	76%	100%	97%	98%
NM_120505	AtPAL3	81%	83%	76%	77%	77%
AC009400	AtPAL4	73%	99%	76%	85%	86%

**Table 3:** *Glycine max *CAD contig comparisons. Tallies of first, second, and third position differences between contigs. Parentheses count gaps as differences. No gaps exist if numbers inside and out are the same. NS means bl2seq returned "no significant similarity." NO means bl2seq returned an alignment but the ORFs did not overlap.

	Contig5	Contig7	Contig8	Contig11	Contig12	Contig15	Contig16	Contig17
	
Contig2	NS	NS	64(64) 44(44) 117(117)	NS	NS	NS	NS	NS
Contig5	***	NS	NS	NS	NS	7(7) 11(11) 38(38)	NS	NS
Contig7	***	***	NS	NS	8(8) 5(5) 28(28)	NS	NS	NS
Contig8	***	***	***	NS	NS	NS	NS	NS
Contig11	***	***	***	***	NS	NS	NS	23(23) 8(8) 27(27)
Contig12	***	***	***	***	***	NS	NS	NS
Contig15	***	***	***	***	***	***	NS	NS
Contig16	***	***	***	***	***	***	***	NS

**Table 4:** *Glycine max *UniGene cluster Gma.9010 contig comparisons. Tallies of first, second, and third position differences between contigs. Parentheses count gaps as differences. No gaps exist if numbers inside and out are the same. NS means bl2seq returned "no significant similarity." NO means bl2seq returned an alignment but the ORFs did not overlap.

	Contig1b	Contig2a	Contig2b
	
Contig1a	0(0) 0(0) 0(0)	15(17) 6(8) 6(8)	15(17) 6(8) 6(8)
Contig1b	******	6(7) 6(7) 15(16)	6(7) 6(7) 15(16)
Contig2a	******	******	0(0) 0(0) 0(0)
